# Diagnostic Challenges in the Management of Aortic Valve Stenosis and the Role of Imaging: A Narrative Review

**DOI:** 10.3390/jcm14041231

**Published:** 2025-02-13

**Authors:** Dimitrios Karelas, Evangelos Tatsis, Dimitrios Oikonomidis, Constantinos Hristou Papadopoulos

**Affiliations:** 2nd Cardiology Department, “Korgialenio–Benakio” Red Cross Hospital, 11526 Athens, Greece; tatsisevangelos@gmail.com (E.T.); dimitris_oikon@yahoo.gr (D.O.)

**Keywords:** aortic valve stenosis, echocardiography, low-flow, low-gradient aortic stenosis, imaging

## Abstract

Aortic valve stenosis (AS) is a prevalent and progressive valvular disease that poses significant diagnostic challenges, particularly in low-flow, low-gradient (LF-LG) states. Accurate assessment of AS severity is crucial for timely intervention and improved clinical outcomes. This narrative review critically evaluates the limitations of conventional echocardiographic techniques and explores the role of multimodal imaging—including advanced echocardiography, computed tomography (CT), and cardiac magnetic resonance (CMR)—in enhancing diagnostic accuracy. Special emphasis is placed on the unique challenges of LF-LG AS, where standard Doppler-derived assessments may misclassify disease severity, necessitating a more integrative diagnostic approach. By addressing these key diagnostic uncertainties and proposing a multimodal framework for improved assessment, this review provides a comprehensive update on best practices in AS evaluation, with the goal of optimizing clinical decision making and patient outcomes.

## 1. Introduction

Aortic valve stenosis (AS) is one of the most common and severe valvular heart diseases, particularly in elderly populations, with an estimated prevalence of more than 5% in individuals over 75 years of age [1]. It is characterized by progressive narrowing of the aortic valve, which leads to the obstruction of blood flow from the left ventricle (LV) to the aorta, ultimately causing left ventricular hypertrophy (LVH), heart failure, and sudden cardiac death if left untreated [2]. Accurate and timely diagnosis is crucial for the management of AS, especially since early intervention can significantly improve outcomes, including survival [3]. However, diagnosing AS remains challenging due to technical limitations in imaging modalities, measurement errors, and the heterogeneous nature of the disease [4].

Among the most diagnostically complex subsets of AS is low-flow, low-gradient (LF-LG) AS. Traditional echocardiographic parameters, including transvalvular pressure gradients and aortic valve area (AVA), may yield discordant findings, complicating clinical decision making. This diagnostic uncertainty underscores the need for a multimodal imaging approach that integrates transthoracic echocardiography (TTE), transesophageal echocardiography (TOE), dobutamine stress echocardiography (DSE), CT-based calcium scoring, and cardiac magnetic resonance (CMR) [5]. The therapeutic implications of misclassifying AS severity are substantial, as both under- and overestimation of severity can lead to suboptimal treatment strategies, either delaying necessary interventions or exposing patients to unnecessary procedural risks.

This review aims to provide a comprehensive assessment of the current diagnostic landscape of AS, with a particular focus on LF-LG AS. By evaluating the strengths and limitations of existing imaging modalities and highlighting the potential of an integrative diagnostic strategy, this review seeks to advance the clinical approach to AS evaluation and management.

## 2. Transthoracic Echocardiography (TTE) in AS

TTE remains the gold standard for diagnosing AS due to its non-invasive nature, widespread availability, and ability to provide comprehensive hemodynamic data. Aortic valve (AV) morphology (thickness, calcification, mobility), AS severity (mild, moderate, severe), and AS consequences in ventricular and atrial volumes and function as well as in heart hemodynamics are the key parameters evaluated principally by TTE and in cases with diagnostic difficulties by CT and CMR [6].

Doppler echocardiography remains the primary technique for the estimation of AVA and the quantification of the severity of AS, primarily through the assessment of transvalvular flow velocities and pressure gradients. The simplified Bernoulli equation is often applied to calculate the pressure gradient (Δ*P* = 4*V*^2^, where *V* is the peak velocity of blood flow through the valve), a key determinant in the grading of AS severity [7]. AS severity can be stratified based on Doppler-derived parameters as outlined below.

- Mild AS: mean gradient < 20 mmHg, peak velocity 2–2.9 m/s, AVA > 1.5 cm^2^.
- Moderate AS: mean gradient 20–40 mmHg, peak velocity 3–3.9 m/s, AVA 1–1.5 cm^2^.
- Severe AS: mean gradient ≥ 40 mmHg, peak velocity ≥ 4 m/s, AVA ≤ 1 cm^2^.

In addition, very severe AS may present with mean gradients ≥ 60 mmHg and peak velocities ≥ 5 m/s. It is important to note that Doppler gradients are rarely overestimated, and a high velocity with a calcified valve is typically diagnostic of severe AS, irrespective of AVA [8].

However, technical issues and potential measurement errors limit the diagnostic value of TTE, particularly in patients with a low-flow state.

### 2.1. Technical Limitations and Measurement Errors

Despite its broad utility, TTE has several inherent limitations. Operator dependency is a significant concern, as the quality and accuracy of the images are highly reliant on the experience and skill of the sonographer. Doppler beam misalignment is another critical limitation, particularly when obtaining peak velocity measurements. The accuracy of these measurements is critically dependent on the alignment of the Doppler beam with the direction of blood flow. Inadequate alignment, especially from apical views, can lead to underestimation of both peak velocity and pressure gradients, resulting in the misclassification of AS severity [9]. Studies suggest that Doppler alignment errors can occur in up to 20% of cases, particularly when apical windows are suboptimal, or when alternative imaging windows (e.g., suprasternal or right parasternal views) are not employed [10]. To mitigate these issues, multi-view imaging, including apical, suprasternal, and right parasternal windows, is essential to accurately capture flow velocities and avoid underestimating the pressure gradients [11]. Doppler flow measurements are also sensitive to the positioning of the pulsed-wave Doppler cursor, and placing it too close to the valve can result in capturing flow acceleration from the obstruction rather than actual left ventricular outflow tract (LVOT) flow, leading to inaccurate AVA calculation and dimensionless index (DI) [12].

Moreover, Doppler imaging can struggle to account for pressure recovery, a phenomenon in which part of the kinetic energy of the transvalvular jet is recovered as pressure downstream of the valve [13]. Pressure recovery is particularly common in patients with small aortic roots, such as women, and can lead to discrepancies between Doppler and catheter-based gradient measurements. In such cases, Doppler may overestimate the severity of stenosis, particularly if flow acceleration is captured too close to the aortic valve, leading to an exaggerated gradient. In patients with heart rhythm disorders, such as atrial fibrillation, continuous and pulse-wave Doppler parameters should be estimated as the mean value of at least five consecutive cardiac cycles at relatively constant R-R intervals [14].

**Figure 1:**
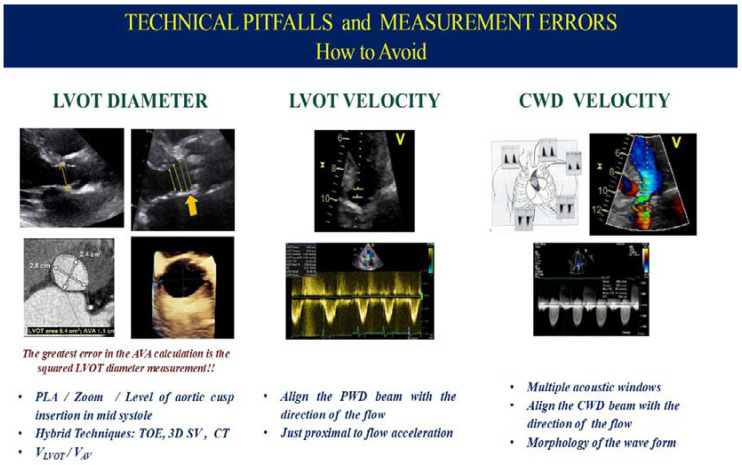
Technical limitations, measurement errors, and how to avoid them during an echocardiographic study for the evaluation of aortic valve stenosis.

Another limitation is Doppler’s reliance on geometric assumptions for flow quantification, particularly in the measurement of stroke volume (SV) through the LVOT. The Doppler method assumes a circular cross-section of the LVOT; however, in reality, the LVOT is often elliptical, leading to errors in the calculation of SV and, subsequently, AVA [15]. Small inaccuracies in measuring the LVOT diameter can be magnified due to its squared contribution in the continuity equation, potentially leading to significant AVA underestimation. This is especially problematic in patients with complex AV anatomy, such as those with bicuspid valves, where standard two-dimensional imaging may not fully capture the elliptical geometry of the LVOT [16] (Figure 1).

In response to these limitations, 3D echocardiography has emerged as a valuable advancement, offering superior visualization of aortic valve morphology and improved accuracy in measuring both AVA and LVOT dimensions [17]. By enhancing spatial resolution and reducing interobserver variability, 3D echocardiography addresses many of the shortcomings associated with traditional 2D imaging. However, despite its potential, 3D echocardiography is not yet widely available in routine clinical practice due to higher costs, the need for specialized software, and the requirement for additional operator expertise [18]. Limited accessibility, particularly in smaller centers, remains a barrier to its broader adoption. As technology advances and accessibility improves, 3D echocardiography may become a more integral part of the diagnostic toolkit for managing aortic stenosis.

Strategies to mitigate the aforementioned issues include the use of multiple imaging windows, improved training programs for sonographers, and emerging AI-driven automation tools that enhance reproducibility [19].

### 2.2. Diagnostic Challenges in Low-Flow, Low-Gradient (LF-LG) AS

In patients with preserved ejection fraction (EF), the presence of a low transvalvular mean gradient (MG) < 40 mmHg despite an AVA ≤ 1 cm^2^ can be explained by several mechanisms. To begin with, measurement artifacts or errors in echocardiographic assessment, particularly in AVA measurement, may lead to the underestimation of AS severity, resulting in misclassification in approximately 50% of cases. Secondly, true LF-LG severe AS may occur, where a genuinely severe stenosis is accompanied by a low gradient due to a reduction in SV, even in the presence of normal EF. Thirdly, normal-flow, low-gradient severe AS can arise from discrepancies in guideline cut-points, where an AVA of 1 cm^2^ may be associated with an MG of approximately 30 mmHg under normal flow conditions [20].

Classical LF-LG AS is characterized by a reduced EF (<50%), a low stroke volume index (SVi) (<35 mL/m^2^), and an MG <40 mmHg. This phenotype often results from impaired myocardial contractility, necessitating dobutamine stress echocardiography (DSE) to differentiate between true severe AS and pseudo-severe AS^20^. However, this method is limited in cases of fixed low flow due to preload- or afterload-related constraints.

Paradoxical LF-LG AS, by contrast, presents with a preserved EF (≥50%) but reduced SV due to a small LV cavity, hypertensive afterload, or impaired diastolic filling. It is more frequently observed in elderly or hypertensive patients, where the combined effects of systemic hypertension and AS (valvuloarterial impedance) reduce transvalvular flow despite maintained systolic function [21].

A normal EF does not necessarily indicate normal cardiac output, as low SV can persist despite normal contractility. Contributing factors include hypertension, which increases total afterload and can lead to stroke volume reduction even with preserved EF. In up to 30% of cases with high afterload, normalization of systemic pressure may falsely suggest milder AS severity. Mitral valve disease and atrial fibrillation can impair ventricular filling, resulting in reduced forward flow. Bradycardia can cause stroke volume to be prolonged rather than increased, thereby reducing true transvalvular flow. Additionally, cardiac amyloidosis, identified in 15% of elderly patients with low-gradient AS, can contribute to impaired myocardial mechanics [22].

The limitations of DSE in paradoxical LF-LG AS necessitate alternative approaches, such as nitroprusside infusion or exercise testing, to better define stenosis severity under dynamic conditions. Invasive hemodynamic assessment with careful afterload reduction can be particularly useful in borderline cases.

Despite initial concerns about lower gradient severity, truly severe LF-LG AS is associated with poor prognosis and limited response to medical therapy. AV replacement (AVR) remains the definitive intervention, improving cardiac output reserve and long-term survival.

## 3. Advanced Echocardiographic Techniques

### 3.1. Dobutamine Stress Echocardiography

DSE is a valuable tool in the evaluation of LF-LG AS, differentiating between true severe and pseudo-severe stenosis.

**Figure 2:**
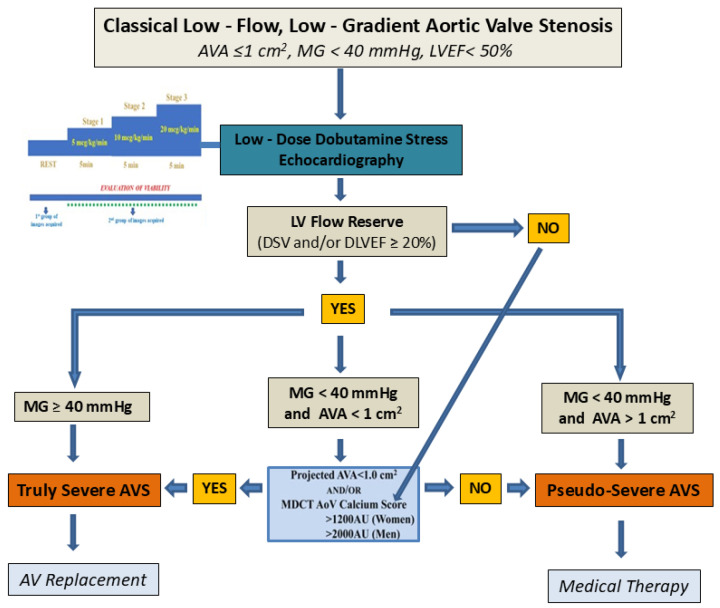
Dobutamine stress echocardiography in patients with low-flow, low-gradient aortic stenosis state and reduced ejection fraction.

In LF-LG AS with reduced EF, DSE is often used to increase SV and clarify the diagnosis (Figure 2). Three distinct responses are typically observed during dobutamine infusion:

**True severe AS**: the SV and MG increase (SV > 15–20% and MG > 40 mmHg), but the AVA remains ≤1 cm^2^.

**Pseudo-severe AS**: the SV increases >15–20%, the MG remains <40 mmHg, and the AVA increases >1 cm^2^.

**No contractile reserve**: The SV does not increase significantly (<15–20%), and the AVA and MG remain unchanged. These patients often have the worst prognosis [23].

In case that the SV increases >15–20%, the MG remains <40 mmHg, and the AVA also remains >1 cm^2^, the projected AVA and/or CT calcium score are used for the differentiation between severe and pseudo-severe AS. Projected AVA is defined as the AVA at a standardized normal flow rate and provides an estimate of what would be the AVA if the patient reached a normal flow rate with DSE. Projected AVA is estimated by a complex equation and subject to the potential errors of LVOT diameter and LVOT VTI estimation [24].

Useful as it is, DSE requires pharmacological stress, which may not be suitable for all patients, and accurate interpretation is highly dependent on operator expertise. The technique’s limited availability in non-tertiary centers further complicates its routine use, necessitating greater efforts to standardize its implementation [25].

AVS: aortic valve stenosis, MG: mean gradient, AVA: aortic valve area, LVEF: left ventricular ejection fraction, DSV: change in stroke volume, DLVEF: change in left ventricular ejection fraction, MDCT: multidetector computed tomography, AoV: aortic valve, AU: Agatston units.

### 3.2. Exercise Stress Echocardiography

Exercise stress echocardiography (ESE) is an important non-invasive tool for the evaluation of AS in asymptomatic patients, particularly by unmasking the symptoms, assessing functional capacity, and evaluating the hemodynamic response of the AV and LV during exercise [26]. An abnormal exercise response or echocardiographic findings of poor prognosis during ESE may affect the timing of interventional therapy in patients with severe asymptomatic AS. ESE indices of poor prognosis in high-gradient AS are the following [27]:

- AV MG increases by >18–20 mmHg, associated with a 3.8-fold higher risk of cardiac events.
- Pulmonary artery systolic pressure (PASP) increases at >60 mmHg, associated with a 2-fold higher risk of a cardiac event at 3-year follow-up.
- Worsening in LVEF or a slight increase in LVEF < 5%.

Despite its benefits, ESE can sometimes be technically demanding due to patient-related factors such as poor acoustic windows or limited exercise tolerance.

### 3.3. Transesophageal Echocardiography (TOE)

TOE plays a crucial role in the evaluation and management of AS, particularly in cases where TTE is inadequate or inconclusive. Its ability to provide high-resolution, close-up images of the AV and surrounding structures makes it an essential tool in diagnosing complex AS cases. TOE is especially valuable when TTE images are compromised by poor acoustic windows, as seen in patients with obesity, chronic obstructive pulmonary disease, or other anatomical challenges.

One of the primary roles of TOE in AS is its use in preoperative planning and intraoperative guidance during AVR procedures, both surgical (SAVR) and transcatheter (TAVR). The detailed anatomical information provided by TOE allows for precise anatomical measurement of the AVA, better visualization of valve leaflet morphology, and the detection of associated conditions such as aortic root dilation or concomitant mitral valve disease. Occasionally, TOE can be helpful in guiding valve deployment during TAVR procedures, ensuring optimal positioning and reducing the risk of complications like paravalvular leaks [28].

TOE is also particularly effective in identifying bicuspid aortic valve (BAV) anatomy, which can be challenging to assess with TTE due to its complex structure and flow dynamics [29]. This is important, as BAV is a common congenital cause of AS, and its accurate identification is critical for surgical planning. TOE’s proximity to the heart allows for superior imaging, ensuring that subtle anatomical features, such as leaflet calcification and aortic root involvement, are captured in detail. Direct AVA planimetry might be difficult when severe calcification of the aortic cusps is present. TOE planimetry appears to overestimate the AVA compared to continuity equation; therefore, measurements slightly above severe AS cut-off values should be interpreted with caution. Possible reasons for this discrepancy are the calculation of the effective orifice area (instead of the anatomical) with the continuity equation as well as the movement of the aortic annulus along its long-axis direction during the cardiac cycle, reducing the accuracy of planimetry at the tips of the AV. Three-dimensional TOE may ameliorate this discrepancy when the image quality is adequate.

Although TOE is less commonly used for quantifying hemodynamics compared to TTE, it still provides valuable insight in certain cases. For instance, TOE can be used when Doppler alignment is difficult in TTE, offering more reliable estimates of transvalvular flow and pressure gradients in specific imaging planes [19]. Furthermore, TOE is invaluable in assessing LF-LG AS, where TTE may not provide clear differentiation between true severe and pseudo-severe stenosis.

### 3.4. Myocardial Deformation—Strain 

Myocardial deformation, particularly through global longitudinal strain (GLS), has become an important tool in the assessment of AS [30]. Strain imaging allows for the early detection of subclinical left ventricular dysfunction before a noticeable decrease in LVEF occurs [31]. GLS is especially sensitive to changes in the subendocardial fibers, which are often affected early in the course of AS due to pressure overload, hypertrophy, and subsequent fibrosis. Given its prognostic significance in AS, GLS could be integrated into standard diagnostic pathways. In patients with moderate-to-severe AS, particularly those with preserved LVEF, the routine assessment of GLS provides the early identification of myocardial dysfunction, allowing for timely intervention. Several studies have demonstrated that impaired GLS is a strong predictor of adverse outcomes in AS, including worse survival and increased risk of heart failure hospitalization [32,33]. The strong relationship between LV GLS and all-cause mortality in asymptomatic patients with AS and preserved LVEF is demonstrated in a recent meta-analysis where an impaired LV GLS measured by speckle tracking imaging less than 15% in asymptomatic patients with significant AS and normal LVEF is associated with reduced survival [34]. In asymptomatic severe AS, the presence of impaired GLS may justify earlier consideration for AV intervention, particularly in conjunction with other risk factors such as elevated natriuretic peptides (NPs) or exercise-induced symptoms [35]. Incorporating GLS with CMR-based fibrosis assessment could further enhance risk stratification, particularly in patients with discordant AS severity grading. While current guidelines predominantly emphasize symptom status and conventional echocardiographic measures, the expanding evidence base supports the role of strain imaging in refining risk stratification and guiding the optimal timing of intervention in severe AS.

## 4. Other Imaging Modalities

### 4.1. Computed Tomography

CT has become an important tool in the diagnostic assessment of AS, particularly in its ability to provide detailed anatomical information and quantify valve calcification. CT calcium scoring is particularly valuable in cases where there is discordance between clinical findings and echocardiographic measurements, such as in patients with LF-LG AS [36]. High calcium scores—typically above 2000 Agatston units in men and 1200 in women—are strongly indicative of severe AS, even when echocardiographic findings suggest a moderate disease state. CT also offers precise measurement of AV anatomy and the LVOT, helping to reduce errors that may occur with two-dimensional imaging techniques like echocardiography [37]. Moreover, CT is the gold standard tool in order to assess the anatomy of the aortic root, valve annulus, and iliofemoral system, which are critical for planning interventions, such as TAVR. Despite its diagnostic advantages, concerns regarding radiation exposure, contrast-induced nephropathy, and cost remain significant barriers. While newer low-dose protocols have mitigated some of these concerns, access to cardiac CT remains limited in many institutions, preventing its widespread integration into routine AS assessment.

### 4.2. Cardiac Magnetic Resonance

CMR is an increasingly utilized modality in the diagnosis and management of AS, offering significant advantages in the assessment of both valve anatomy and myocardial function. One of the most unique contributions of CMR is its ability to quantify myocardial fibrosis, which has important prognostic implications in patients with AS [38]. Fibrosis, detected via late gadolinium enhancement (LGE), can indicate myocardial stress and predict adverse outcomes, even in patients who may not yet exhibit significant symptoms. In addition to its superior soft tissue characterization, CMR provides highly accurate measurements of AVA and left ventricular volumes without the geometric assumptions required in echocardiography [39]. CMR also offers insights into flow patterns and the severity of AS without the ionizing radiation associated with CT. However, MRI is limited by its relatively high cost, technical complexity, specialized radiological expertise, long scan times, and the need for patient cooperation during scanning. Additionally, limited availability in non-specialized centers restricts its use to select patient populations, preventing broader adoption in routine AS evaluation. Despite these challenges, it is emerging as a valuable tool in cases where echocardiography provides inconclusive results, particularly in low-flow, low-gradient AS.

## 5. Discussion

AS presents significant diagnostic challenges, particularly given the reliance on echocardiography as the primary diagnostic tool. While TTE is the gold standard for initial assessment, it has limitations in certain clinical contexts. Issues such as operator dependency, Doppler misalignment, and difficulties in evaluating complex valvular anatomies can lead to underestimation or overestimation of AS severity. The growing recognition of the limitations of echocardiography, particularly in measuring LVOT diameter and assessing low-flow states, emphasizes the importance of integrating multimodal diagnostic approaches.

Combining conventional TTE with novel advanced echocardiographic techniques and other imaging modalities such as cardiac CT and CMR provides a more robust assessment of AS severity. Three-dimensional echocardiography, for instance, offers superior visualization of valve anatomy and function, improving the accuracy of AVA and annular measurements and reducing interobserver variability. Strain imaging, particularly GLS, has proven to be a sensitive marker for detecting early left ventricular dysfunction, even in patients with preserved LVEF.

CT-based calcium scoring presents as a valuable adjunct, particularly in cases with discordant AS severity grading. A high aortic valve calcification burden, exceeding 2000 Agatston units in men and 1200 in women, suggests a high likelihood of truly severe AS and supports early AVR consideration [40]. Similarly, CMR provides essential insights into myocardial fibrosis, as LGE serves as a strong predictor of adverse outcomes. Patients with extensive fibrosis, even if asymptomatic, exhibit a higher risk of progression and may derive benefit from earlier surgical intervention. Exercise stress echocardiography plays a crucial role in uncovering latent symptoms and hemodynamic abnormalities that may not be evident at rest^27^. An exaggerated rise in pulmonary pressures or a significant increase in transvalvular gradients during exercise, exceeding 20 mmHg, identifies a subset of patients with higher risk [41]. Integrating imaging findings with biomarkers, such as NT-proBNP, further refines risk stratification, particularly in borderline cases where intervention decisions remain challenging [42]. By integrating strain imaging and multimodal assessment into standard workflows, clinicians can more precisely identify AS patients at risk of adverse outcomes and refine therapeutic decision making beyond traditional guidelines. A forward-looking approach incorporating innovative imaging strategies will facilitate the development of personalized management strategies for AS, ultimately improving patient prognosis and long-term outcomes.

A summary table of imaging modalities used in the evaluation of AS is provided (Table 1).

### Limitations and Future Perspectives

The assessment and management of AS face significant challenges due to the lack of standardized thresholds for integrating multimodal imaging findings, which complicates risk stratification and treatment decisions. Future research should prioritize refining imaging methodologies and establishing consensus-driven protocols that systematically incorporate echocardiography, CT, and CMR. These advancements will help address current limitations, such as variability in CT-based calcium scoring, CMR fibrosis quantification, and exercise stress echocardiography, ensuring more consistent and accurate AS evaluation across institutions.

AI holds significant promise in overcoming these challenges. AI-driven algorithms can automate critical measurements, such as AVA and left LVOT dimensions, reducing operator dependency and improving reproducibility [43,44]. Machine learning models may enable more precise, individualized risk assessment, optimizing intervention timing. Additionally, AI-powered tools for GLS measurement can enhance the detection of subclinical myocardial dysfunction, further refining risk stratification.

To validate these innovations, long-term prospective studies are essential to assess the impact of advanced imaging and AI-guided decision making on clinical outcomes. Randomized trials comparing imaging-guided intervention strategies to conventional approaches could optimize patient selection for early valve replacement, particularly in asymptomatic or LF-LG AS. Standardized protocols for multimodal imaging, combined with AI-enhanced analytics, have the potential to transform AS management by enabling a more personalized, patient-centered approach.

## 6. Conclusions

In conclusion, AS is a valvular disease with significant diagnostic challenges, particularly in LF-LG states. Thorough clinical evaluation, optimal use of TTE, and the integration of advanced imaging technologies, AI-driven tools, and standardized protocols will address current limitations in AS assessment, improving diagnostic accuracy, risk stratification, and therapeutic decision making. By fostering interdisciplinary collaboration and prioritizing innovation, clinicians can enhance long-term patient outcomes and optimize resource utilization in the management of AS.

## Figures and Tables

**Table 1:** Summary of imaging modalities in AS.

Imaging Modality	Strengths	Weaknesses	Clinical Applications
TTE	-Non-invasive, widely available. -Comprehensive hemodynamic data. -Gold standard for AS diagnosis.	-Operator-dependent. -Poor acoustic windows. -Doppler misalignment may underestimate gradients.	-First-line diagnostic tool. -Assesses valve morphology, flow velocity, and gradients.
TOE	-Superior image resolution. -Detailed valve anatomy. -Ideal for intraoperative guidance.	-Semi-invasive (requires sedation). -Limited in esophageal pathology.	-Used when TTE is suboptimal. -Preoperative planning. -Evaluates complex anatomy.
DSE	-Assesses AS severity in LF-LG AS with reduced LVEF. -Differentiates true vs. pseudo-severe AS.	-Requires dobutamine. -Limited to patients with cardiac reserve. -Complex to perform.	-Evaluates contractile reserve. -Guides surgical decisions in LF-LG AS.
ESE	-Unmasks symptoms in asymptomatic AS. -Dynamic hemodynamic assessment. -Low cost.	-Technically challenging. -Limited by exercise tolerance. -Less precise than DSE.	-Assesses exercise tolerance and prognosis. -Guides intervention timing.
GLS	-Detects subclinical LV dysfunction. -Predicts adverse outcomes (HF, mortality). -Non-invasive.	-Requires specialized software. -Interobserver variability. -Limited availability.	-Risk stratification. -Guides intervention in asymptomatic AS with preserved LVEF.
CT	-Excellent anatomical assessment. -Precise AVA measurement. -Calcium scoring reclassifies severity.	-Radiation exposure. -Limited hemodynamic data. -May require contrast.	-Preoperative planning for TAVR. -Assesses valve calcification. -Clarifies severity in borderline cases.
CMR	-Accurate volumetric measurements. -Quantifies myocardial fibrosis (LGE). -No radiation.	-High cost, limited availability. -Long scan times. -Contraindicated in some implants.	-Assesses myocardial function and fibrosis. -Used when echocardiography is inconclusive.

## Data Availability

Data are contained within the article.

## Abbreviations

AS: aortic stenosis; AV: aortic valve; AVA: aortic valve area; BAV: bicuspid aortic valve; CT: computed tomography; CMR: cardiac magnetic resonance; DSE: dobutamine stress echocardiography; ESE: exercise stress echocardiography; LF-LG AS: low-flow, low-gradient AS; LGE: late gadolinium enhancement; LVOT: left ventricular outflow tract; LV: left ventricle; LVH: left ventricular hypertrophy; SAVR: surgical aortic valve replacement; SV: stroke volume; TAVR: transcatheter aortic valve replacement; TOE: transesophageal echocardiography; TTE: transthoracic echocardiography.

## References

[1] Coffey S., Cairns B.J., Iung B. (2016). The modern epidemiology of heart valve disease. Heart.

[2] Joseph J., Naqvi S.Y., Giri J., Goldberg S. (2017). Aortic Stenosis: Pathophysiology, Diagnosis, and Therapy. Am. J. Med..

[3] Boskovski M.T., Gleason T.G. (2021). Current Therapeutic Options in Aortic Stenosis. Circ. Res..

[4] Sherwood M.W., Kiefer T.L. (2017). Challenges in Aortic Valve Stenosis: Low-Flow States Diagnosis, Management, and a Review of the Current Literature. Curr. Cardiol. Rep..

[5] Gaznabi S., Miranda J., Lorenzatti D., Piña P., Balasubramanian S.S., Desai D., Desai A., Ho E.C., Scotti A., Gongora C.A. (2024). Multimodality Imaging in Aortic Stenosis: Beyond the Valve—Focusing on the Myocardium. Cardiol. Clin..

[6] González-García A., Pazos-López P., Calvo-Iglesias F.E., Matajira-Chía T.M., Bilbao-Quesada R., Blanco-González E., González-Ríos C., Castiñeira-Busto M., Barreiro-Pérez M., Íñiguez-Romo A. (2024). Diagnostic Challenges in Aortic Stenosis. J. Cardiovasc. Dev. Dis..

[7] Franke B., Weese J., Waechter-Stehle I., Brüning J., Kuehne T., Goubergrits L. (2020). Towards improving the accuracy of aortic transvalvular pressure gradients: Rethinking Bernoulli. Med. Biol. Eng. Comput..

[8] Vahanian A., Beyersdorf F., Praz F., Milojevic M., Baldus S., Bauersachs J., Capodanno D., Conradi L., De Bonis M., De Paulis R. (2022). 2021 ESC/EACTS Guidelines for the management of valvular heart disease. Eur. Heart J..

[9] Zoghbi W.A., Adams D., Bonow R.O., Enriquez-Sarano M., Foster E., Grayburn P.A., Hahn R.T., Han Y., Hung J., Lang R.M. (2017). Recommendations for Noninvasive Evaluation of Native Valvular Regurgitation: A Report from the American Society of Echocardiography Developed in Collaboration with the Society for Cardiovascular Magnetic Resonance. J. Am. Soc. Echocardiogr..

[10] Baumgartner H., Hung J., Bermejo J., Chambers J.B., Edvardsen T., Goldstein S., Lancellotti P., LeFevre M., Miller F., Otto C.M. (2017). Recommendations on the echocardiographic assessment of aortic valve stenosis: A focused update from the European Association of Cardiovascular Imaging and the American Society of Echocardiography. Eur. Heart J. Cardiovasc. Imaging.

[11] De Monchy C.C., Lepage L., Boutron I., Leye M., Detaint D., Hyafil F., Brochet E., Iung B., Vahanian A., Messika-Zeitoun D. (2009). Usefulness of the right parasternal view and non-imaging continuous-wave Doppler transducer for the evaluation of the severity of aortic stenosis in the modern area. Eur. J. Echocardiogr..

[12] Lancellotti P., Tribouilloy C., Hagendorff A., Moura L., Popescu B.A., Agricola E., Monin J.-L., Pierard L.A., Badano L., Zamorano J.L. (2010). European Association of Echocardiography. European Association of Echocardiography recommendations for the assessment of valvular regurgitation. Part 1: Aortic and pulmonary regurgitation (native valve disease). Eur. J. Echocardiogr..

[13] Levine R.A., Jimoh A., Cape E.G., McMillan S., Yoganathan A.P., Weyman A.E. (1989). Pressure recovery distal to a stenosis: Potential cause of gradient “overestimation” by Doppler echocardiography. J. Am. Coll. Cardiol..

[14] Kotecha D., Mohamed M., Shantsila E., Popescu B.A., Steeds R.P. (2017). Is echocardiography valid and reproducible in patients with atrial fibrillation? A systematic review. Europace.

[15] Orwat S., Kaleschke G., Kerckhoff G., Radke R., Baumgartner H. (2013). Low flow, low gradient severe aortic stenosis: Diagnosis, treatment and prognosis. EuroIntervention.

[16] Liu S., Churchill J., Hua L., Zeng X., Rhoades V., Namasivayam M., Baliyan V., Ghoshhajra B.B., Dong T., Dal-Bianco J.P. (2020). Direct Planimetry of Left Ventricular Outflow Tract Area by Simultaneous Biplane Imaging: Challenging the Need for a Circular Assumption of the Left Ventricular Outflow Tract in the Assessment of Aortic Stenosis. J. Am. Soc. Echocardiogr..

[17] Lang R.M., Badano L.P., Tsang W., Adams D.H., Agricola E., Buck T., Faletra F.F., Franke A., Hung J., De Isla L.P. (2012). EAE/ASE recommendations for image acquisition and display using three-dimensional echocardiography. Eur. Heart J. Cardiovasc. Imaging.

[18] Clavel M.A., Pibarot P. (2014). Assessment of low-flow, low-gradient aortic stenosis: Multimodality imaging is the key to success. EuroIntervention.

[19] Vasile C.M., Iriart X. (2023). Embracing AI: The Imperative Tool for Echo Labs to Stay Ahead of the Curve. Diagnostics.

[20] Clavel M.-A., Magne J., Pibarot P. (2016). Low-gradient aortic stenosis. Eur. Heart J..

[21] Mantha Y., Futami S., Moriyama S., Hieda M. (2021). Valvulo-Arterial Impedance and Dimensionless Index for Risk Stratifying Patients With Severe Aortic Stenosis. Front. Cardiovasc. Med..

[22] Bistola V., Parissis J., Foukarakis E., Valsamaki P.N., Anastasakis A., Koutsis G., Efthimiadis G., Kastritis E. (2021). Practical recommendations for the diagnosis and management of transthyretin cardiac amyloidosis. Heart Fail Rev..

[23] Rizzello V. (2021). Moderate gradient severe aortic stenosis: Diagnosis, prognosis and therapy. Eur. Heart J. Suppl..

[24] Namasivayam M., He W., Churchill T.W., Capoulade R., Liu S., Lee H., Danik J.S., Picard M.H., Pibarot P., Levine R.A. (2020). Transvalvular Flow Rate Determines Prognostic Value of Aortic Valve Area in Aortic Stenosis. J. Am. Coll. Cardiol..

[25] Clavel M.-A., Burwash I.G., Mundigler G., Dumesnil J.G., Baumgartner H., Bergler-Klein J., Sénéchal M., Mathieu P., Couture C., Beanlands R. (2010). Validation of conventional and simplified methods to calculate projected valve area at normal flow rate in patients with low flow, low gradient aortic stenosis: The multicenter TOPAS (True or Pseudo Severe Aortic Stenosis) study. J. Am. Soc. Echocardiogr..

[26] Clavel M.-A., Ennezat P.V., Maréchaux S., Dumesnil J.G., Capoulade R., Hachicha Z., Mathieu P., Bellouin A., Bergeron S., Meimoun P. (2013). Stress echocardiography to assess stenosis severity and predict outcome in patients with paradoxical low-flow, low-gradient aortic stenosis and preserved LVEF. JACC Cardiovasc. Imaging.

[27] Onishi T., Sengoku K., Ichibori Y., Mizote I., Maeda K., Kuratani T., Sawa Y., Sakata Y. (2018). The role of echocardiography in transcatheter aortic valve implantation. Cardiovasc. Diagn. Ther..

[28] Bhushan S., Huang X., Li Y., He S., Mao L., Hong W., Xiao Z. (2022). Paravalvular Leak After Transcatheter Aortic Valve Implantation Its Incidence, Diagnosis, Clinical Implications, Prevention, Management, and Future Perspectives: A Review Article. Curr. Probl. Cardiol..

[29] Afzal S., Piayda K., Maier O., Goh S., Hellhammer K., Cramer M., Bönner F., Polzin A., Nijhof N., Kelm M. (2020). Current and Future Aspects of Multimodal Imaging, Diagnostic, and Treatment Strategies in Bicuspid Aortic Valve and Associated Aortopathies. J. Clin. Med..

[30] Meredith T., Roy D., Hayward C., Feneley M., Kovacic J., Muller D., Namasivayam M. (2024). Strain Assessment in Aortic Stenosis: Pathophysiology and Clinical Utility. J. Am. Soc. Echocardiogr..

[31] Dahl J.S., Magne J., Pellikka P.A., Donal E., Marwick T.H. (2019). Assessment of Subclinical Left Ventricular Dysfunction in Aortic Stenosis. JACC Cardiovasc. Imaging.

[32] Vollema E.M., Sugimoto T., Shen M., Tastet L., Ng A.C.T., Abou R., Marsan N.A., Mertens B., Dulgheru R., Lancellotti P. (2018). Association of Left Ventricular Global Longitudinal Strain with Asymptomatic Severe Aortic Stenosis: Natural Course and Prognostic Value. JAMA Cardiol..

[33] Vollema E.M., Amanullah M.R., Prihadi E.A., Ng A.C.T., van der Bijl P., Sin Y.K., Marsan N.A., Ding Z.P., Généreux P., Leon M.B. (2020). Incremental value of left ventricular global longitudinal strain in a newly proposed staging classification based on cardiac damage in patients with severe aortic stenosis. Eur. Heart J. Cardiovasc. Imaging.

[34] Magne J., Cosyns B., Popescu B.A., Carstensen H.G., Dahl J., Desai M.Y., Kearney L., Lancellotti P., Marwick T.H., Sato K. (2019). Distribution and Prognostic Significance of Left Ventricular Global Longitudinal Strain in Asymptomatic Significant Aortic Stenosis: An Individual Participant Data Meta-Analysis. JACC Cardiovasc. Imaging.

[35] Lancellotti P., Magne J., Dulgheru R., Clavel M.-A., Donal E., Vannan M.A., Chambers J., Rosenhek R., Habib G., Lloyd G. (2018). Outcomes of Patients with Asymptomatic Aortic Stenosis Followed Up in Heart Valve Clinics. JAMA Cardiol..

[36] Scalia I.G., Farina J.M., Padang R., Jokerst C.E., Pereyra M., Mahmoud A.K., Naqvi T.Z., Chao C.J., Oh J.K., Arsanjani R. (2023). Aortic Valve Calcium Score by Computed Tomography as an Adjunct to Echocardiographic Assessment-A Review of Clinical Utility and Applications. J. Imaging.

[37] Hell M.M., Steinmann B., Scherkamp T., Arnold M.B., Achenbach S., Marwan M. (2021). Analysis of left ventricular function, left ventricular outflow tract and aortic valve area using computed tomography: Influence of reconstruction parameters on measurement accuracy. Br. J. Radiol..

[38] Bing R., Cavalcante J.L., Everett R.J., Clavel M.A., Newby D.E., Dweck M.R. (2019). Imaging and Impact of Myocardial Fibrosis in Aortic Stenosis. JACC Cardiovasc. Imaging.

[39] De Rubeis G., Galea N., Ceravolo I., Dacquino G.M., Carbone I., Catalano C., Francone M. (2019). Aortic valvular imaging with cardiovascular magnetic resonance: Seeking for comprehensiveness. Br. J. Radiol..

[40] Clavel M.-A., Pibarot P., Messika-Zeitoun D., Capoulade R., Malouf J., Aggarwal S.R., Araoz P.A., Michelena H.I., Cueff C., Larose E. (2014). Impact of aortic valve calcification, as measured by MDCT, on survival in patients with aortic stenosis: Results of an international registry study. J. Am. Coll. Cardiol..

[41] Maréchaux S., Hachicha Z., Bellouin A., Dumesnil J.G., Meimoun P., Pasquet A., Bergeron S., Arsenault M., Le Tourneau T., Ennezat P.V. (2010). Usefulness of exercise-stress echocardiography for risk stratification of true asymptomatic patients with aortic valve stenosis. Eur Heart J..

[42] Clavel M.-A., Malouf J., Michelena H.I., Suri R.M., Jaffe A.S., Mahoney D.W., Enriquez-Sarano M. (2014). B-type natriuretic peptide clinical activation in aortic stenosis: Impact on long-term survival. J. Am. Coll. Cardiol..

[43] Zhang J., Gajjala S., Agrawal P., Tison G.H., Hallock L.A., Beussink-Nelson L., Lassen M.H., Fan E., Aras M.A., Jordan C. (2018). Fully Automated Echocardiogram Interpretation in Clinical Practice. Circulation.

[44] Madani A., Arnaout R., Mofrad M., Arnaout R. (2018). Fast and accurate view classification of echocardiograms using deep learning. NPJ Digit Med..

